# Widening the Reach: The Broad Impact of Unguided Self‐Help for Eating Disorders

**DOI:** 10.1002/eat.24460

**Published:** 2025-05-15

**Authors:** Roz Shafran, Sarah J. Egan

**Affiliations:** ^1^ UCL Great Ormond Street Institute of Child Health London UK; ^2^ enAble Institute, Faculty of Health Sciences Curtin University Perth Western Australia Australia; ^3^ Discipline of Psychology, School of Population Health Curtin University Perth Australia

**Keywords:** comorbidity, holistic, session‐by‐session measurement, transdiagnostic

## Abstract

A systematic review and meta‐analysis conducted by Linardon and colleagues on 27 controlled trials using pure self‐help for the prevention and treatment of eating disorders, reported small benefits for co‐occurring difficulties such as anxiety, depression, distress and self‐esteem. The findings were strongest for pre‐selected samples considered at risk or who were symptomatic, and are consistent with literature from other areas indicating that focused interventions have a positive impact on comorbid difficulties. The meta‐analysis raises questions about the optimal approach to address comorbidity both within and beyond pure self‐help. Understanding the wider impact of disorder‐specific approaches compared to transdiagnostic approaches is critical to helping clinicians determine what interventions to use and when. It is notable that CBT interventions across disorders often share treatment techniques and methods to optimize the generalization of learning across difficulties, but such common elements are rarely made explicit. The value of session‐by‐session measurement as an essential tool to guide clinical decision‐making in the context of comorbid difficulties is emphasized. Whilst further work is needed, particularly in clinical samples, the message from Linardon et al.'s meta‐analysis is straightforward—pure self‐help for the prevention and treatment of eating disorders can have a broad impact in improving mental health.

When people talk about the research‐practice gap, the blame is implicitly placed on the clinician. Why aren't clinicians reading the words of wisdom contained in our research papers, implementing the findings and improving outcomes for patients? Occasionally researchers may acknowledge that the research isn't accessible to clinicians or isn't applicable to routine clinical settings and turn to implementation science for answers. Less often do researchers acknowledge that their work is too often simply irrelevant to clinical practice. What do clinicians want to know? We would suggest that many clinicians want help with clinical decision‐making in the face of comorbidity. Why? Because, as described by Linardon et al. (2025) in their meta‐analysis, comorbidity is the rule rather than the exception, with estimates of up to 62% comorbidity between eating disorders and anxiety disorders, and up to 54% between eating disorders and mood disorders (Hambleton et al. 2022).

The options open to clinicians facing such comorbidity are set out in terms of a protocol (Wade et al. 2024), and one of the options is to provide treatment for the eating disorder and determine the impact on the comorbid anxiety and depression. This option is appealing for many reasons, including that the intervention can be delivered in a standard number of sessions without the need for additional sessions. The same principles set out in the Wade et al. (2024) protocol apply to pure (i.e., unguided) and guided self‐help interventions. A strength of the review of Linardon et al. (2025) is that it considers the reality that most individuals with symptoms of eating disorders will never access treatment face‐to‐face or receive a guided self‐help intervention. Examining the pooled efficacy across studies of interventions that are unguided self‐help interventions isthereforecritical to understand. Is it the case that a patient can undergo one self‐help programme for their eating disorder, or do they need to have a series of programmes tackling each of the difficulties (i.e., anxiety and depression)? For many patients, their difficulties are all intertwined, and there is significant variation in individual circumstances with some developing the eating difficulties prior to other mental health problems, but others developing the eating difficulties as a consequence of the other mental health problems.

The Linardon et al. (2025) paper makes such a significant contribution to the field because it informs an understudied yet clinically crucial question about what happens to symptoms of comorbid disorders when focusing on the eating disorder in pure self‐help prevention, early intervention, and treatment programs. The paper included 27 randomized controlled trials with 2388 participants receiving an intervention and 2280 controls. The effects were pooled across selective and indicated prevention programs, along with treatment studies, while universal prevention programmes were excluded. This method makes intuitive sense and is more focused when examining the question of what happens to an individual who receives an intervention who already has elevated risk (e.g., increased weight concerns) or eating disorder symptoms, rather than the general population. The findings are encouraging, with pure self‐help interventions for eating disorders appearing to have small effect size benefits for comorbid depression, anxiety, distress, and self‐esteem. Given prevention studies were pooled together with treatment studies, it is likely that if a future meta‐analysis were conducted restricting only to treatment studies, the effect sizes on comorbid symptoms would be larger. However, most of the studies were of prevention approaches, with fewer examining clinical samples (nine of the 27 studies; 33% overall).

As the authors point out, most studies only included wait‐list control. However, Linardon et al. (2025) did examine within‐group effects within waitlisted participants. Given the sensitivity analyses showing the efficacy of self‐help interventions is not explained by deterioration in waitlist groups, it was concluded that the benefits observed were likely due to the intervention. However, we fully agree that it will be important for future research to go beyond waitlist control designs. Comparison to other active non‐specific interventions is required to offer more rigorous forms of control and account for generic factors such as expectations and attention.

The limitations of the meta‐analysis and findings are well set out, including questions about applicability to clinically diagnosed populations. Such questions reinforce the concern that researchers are not adequately addressing the questions of most importance to clinicians. Why aren't there multiple, high‐quality studies using standardized measures that are specifically designed to help clinicians decide on the best course of action in the face of comorbidity, regardless of whether the intervention is pure self‐help, guided self‐help, or clinician‐led treatment? We suggest this is a serious omission and a rich area to explore in future research.

The tentative conclusion of the systematic review about the benefits of unguided self‐help interventions for the treatment and prevention of eating disorders on comorbidity is consistent with the limited broader literature which shows that a focus on one disorder has benefits on comorbid disorders (e.g., Steele et al. 2018). Such findings also have implications for transdiagnostic approaches that aim to tackle multiple mental health disorders simultaneously by focusing on common maintaining processes. Such transdiagnostic approaches have great appeal, including potential efficiency, improved ease of dissemination and implementation, and possibly improved outcomes. Within eating disorders, transdiagnostic approaches typically refer to different eating disorder diagnoses, but within emotional disorders, approaches such as the unified protocol tackle anxiety and depression (Steele et al. 2018). Interventions targeting transdiagnostic maintaining processes, such as perfectionism, have been shown to improve eating disorder psychopathology as well as anxiety and depression, with the interventions being delivered in a range of formats (Galloway et al. 2022). However, transdiagnostic treatments encompass a heterogeneous set of interventions that vary in scope and application. Some target one core process, while others address multiple; some may be designed for individuals with two co‐occurring diagnoses or applied more broadly across a range of disorders. Approaches range from unified protocols to highly personalized, modular interventions tailored to the individual's specific strengths and difficulties (see Dalgleish et al. 2020 for a detailed and scholarly discussion). Given this variability, questions remain about how best to define, implement, and evaluate transdiagnostic treatments, and enthusiasm for transdiagnostic approaches should not be at the expense of a full understanding of the impact of disorder‐specific approaches on comorbidity.

Further studies are also required that directly compare transdiagnostic approaches to eating disorder specific approaches. Direct comparisons will allow for an understanding of whether an eating disorder focused intervention is sufficient or transdiagnostic approaches may offer further benefits. The meta‐analysis by Linardon et al. (2025) included a wide range of interventions, from dissonance‐based prevention programmes, transdiagnostic approaches (e.g., CBT for perfectionism), and third‐wave approaches (e.g., dialectical behavior therapy, compassion focused, acceptance and commitment therapy), imagery rescripting, and psychoeducation. This can be seen as both a strength as it reflects clinical reality, but also a weakness since the question of whether to have a specific focus on the eating disorder rather than a broader one becomes muddied. As the literature further develops and more studies are available, it would be useful for a future meta‐analysis to compare across intervention approaches, for example, to examine if there are any differences between cognitive‐behavioral eating disorder focused interventions, transdiagnostic approaches, and third‐wave interventions.

What are the mechanisms by which tackling the eating difficulties improves comorbid difficulties? In some cases, it may be obvious. Having eating difficulties impacts mood, anxiety, and wellbeing, and improving the eating disorder symptoms will have a secondary benefit. However, for others, it may be that the eating difficulties are secondary to mood problems or that there are other maintaining processes. This is particularly true of binge‐eating disorder, which was the difficulty targeted in several of the trials, with binge eating often seen as particularly strongly related to mood. Some of the skills in the unguided self‐help interventions, such as problem‐solving, are also included in evidence‐based interventions for depression and anxiety. In many ways it would be more surprising and disappointing if the benefits failed to generalize from one problem area to another.

The article raises questions about improving unguided self‐help interventions as well as guided self‐help interventions and clinician‐led treatment for eating disorders to maximize and generalize benefit. Would including information about applicability to other areas and how to generalize learning help improve the relatively small effect sizes found in the meta‐analysis? If so, how can we use what is known about generalizing learning to determine what information and exercises should be included? The small number of trials included that were of clinical populations (*n* = 9) and low quality of the studies led the authors to appropriately caution against drawing firm conclusions, but it is notable that in the clinical populations, unguided self‐help did not significantly impact depression, anxiety, and self‐esteem. If this is a robust, replicable finding, it raises the question of why it would be the case that the learning from one area is less likely to generalize in the case of clinical populations? Although it may be tempting to say that unguided self‐help may have had less impact on clinical populations in the first place, the literature showing its efficacy and its recommendation as a first‐line treatment for binge‐eating disorder suggests that this is not the case. We would hope there will be stronger data to be able to reach firmer conclusions and better understanding regarding the ability of pure self‐help interventions to reduce broader mental health difficulties associated with clinical eating disorders in the future when a larger number of high‐quality studies are published and can be synthesized.

Many of the studies included in the systematic review were designed to address the eating disorder primarily, rather than to investigate the impact of the intervention on broader mental health disorders. This is an important point because it means that the available data are limited and the research design often suboptimal. If the primary research question of the studies had been to establish the impact on comorbidity, then it is likely that they would have included a range of robust measures of comorbidity and even potentially included session‐by‐session measures of both the eating disorder and comorbid disorders to understand the temporal relationship between changes in one area and the other. The use of session‐by‐session measures is arguably one of the most essential tools when addressing comorbidity (Wade et al. 2024). If a focus on the primary eating disorder has broader benefits, then it is essential to keep track of the broader impact. If there is progress in the eating disorder symptoms but not more broadly, then the intervention can be tailored to optimize the generalizability of learning. If there is progress in the eating disorder and comorbidity, then there is no need for such tailoring. If the comorbid conditions are impeding treatment progress, then that will become clear early on in the course of treatment at a formal review session, and a change of direction in treatment may be warranted.

Such session‐by‐session measurement is often incorporated in self‐help, particularly within apps. However, it is not sufficient to simply present patients with their scores on the measures. Helping patients understand the implications of the scores not just for the eating disorder but more broadly can help people understand the relationship between their difficulties and how intervening in one area may have benefits beyond their eating. Understanding how this can be achieved in unguided self‐help interventions in the future is an important question.

Patients can often be perplexed by clinicians' insistence on separating eating difficulties from the variables assessed in Linardon et al.'s (2025) systematic review. For them, it is all joined up—the eating, the anxiety, the mood, are experienced holistically as a sense of being overwhelmed and distressed. It makes sense, therefore, for unguided self‐help to acknowledge this from the outset and to be explicit about the broader benefits of an intervention programme. It also has implications for the separation of services. Whilst specialisms are important in skilling up clinicians, it does patients a disservice if clinicians who see patients with anxiety and depression are unable to recognize disordered eating and vice versa.

In conclusion, Linardon et al.'s (2025) review synthesizes data that is of importance to people with eating disorder symptoms, clinicians, and services. The wider benefits are currently relatively small, and questions remain about how best to increase them. Nevertheless, it is clear that unguided self‐help holds broad value, offering the potential to positively impact a wide range of psychopathology and expand access to effective interventions.

## Author Contributions

**Roz Shafran:** conceptualization, writing – original draft, writing – review and editing. **Sarah J. Egan:** conceptualization, writing – original draft, writing – review and editing.

## Conflicts of Interest

R.S. and S.E. are authors of a self‐help book Shafran, R., Egan, S. J., and Wade, T. (2019) *Overcoming perfectionism 2nd edition: A self‐help guide using scientifically supported cognitive behavioral techniques*. Robinson Press. S.E. contributed to two of the studies in the systematic review.

## Data Availability

Data sharing not applicable to this article as no datasets were generated or analysed during the current study.

## References

[eat24460-bib-0001] Dalgleish, T. , M. Black , D. Johnston , and A. Bevan . 2020. “Transdiagnostic Approaches to Mental Health Problems: Current Status and Future Directions.” Journal of Consulting and Clinical Psychology 88, no. 3: 179–195. 10.1037/ccp0000482. 32068421 PMC7027356

[eat24460-bib-0002] Galloway, R. , H. Watson , D. Greene , R. Shafran , and S. J. Egan . 2022. “The Efficacy of Randomised Controlled Trials of Cognitive Behaviour Therapy for Perfectionism: A Systematic Review and Meta‐Analysis.” Cognitive Behaviour Therapy 51, no. 2: 170–184. 10.1080/16506073.2021.1952302. 34346282

[eat24460-bib-0003] Hambleton, A. , G. Pepin , A. Le , D. Maloney , S. Touyz , and S. Maguire . 2022. “Psychiatric and Medical Comorbidities of Eating Disorders: Findings From a Rapid Review of the Literature.” Journal of Eating Disorders 10, no. 1: 132. 10.1186/s40337-022-00654-2. 36064606 PMC9442924

[eat24460-bib-0004] Linardon, J. , H. K. Jarman , C. Liu , C. Anderson , Z. McClure , and M. Messer . 2025. “Mental Health Impacts of Self‐Help Interventions for the Treatment and Prevention of Eating Disorders. A Meta‐Analysis.” International Journal of Eating Disorders 58, no. 5: 815–831. 10.1002/eat.24405. 40026263 PMC12067516

[eat24460-bib-0005] Steele, S. J. , T. J. Farchione , C. Cassiello‐Robbins , et al. 2018. “Efficacy of the Unified Protocol for Transdiagnostic Treatment of Comorbid Psychopathology Accompanying Emotional Disorders Compared to Treatments Targeting Single Disorders.” Journal of Psychiatric Research 104: 211–216. 10.1016/j.jpsychires.2018.08.005. 30103069 PMC6219859

[eat24460-bib-0006] Wade, T. D. , R. Shafran , and Z. Cooper . 2024. “Developing a Protocol to Address Co‐Occurring Mental Health Conditions in the Treatment of Eating Disorders.” International Journal of Eating Disorders 57, no. 6: 1291–1299. 10.1002/eat.24008. 37278186

